# Visualization of texture components using *MTEX*

**DOI:** 10.1107/S1600576719014742

**Published:** 2020-02-18

**Authors:** Gennady Rafailov, El’ad N. Caspi, Ralf Hielscher, Eitan Tiferet, Roni Schneck, Sven C. Vogel

**Affiliations:** a Los Alamos Neutron Science Center, Los Alamos National Laboratory, MS H805, Los Alamos, New Mexico 87545, USA; b Materials Department, Nuclear Research Center of the Negev, PO Box 9001, Beer Sheva, 84190, Israel; c Department of Materials Engineering, Ben Gurion University of the Negev, PO Box 653, Beer Sheva, 84105, Israel; d Physics Department, Nuclear Research Center of the Negev, PO Box 9001, Beer Sheva, 84190, Israel; e Fakultät für Mathematik, Technische Universität Chemnitz, Reichenhainer Strasse 39, Chemnitz, 09126, Germany; f Additive Manufacturing Center, Rotem Industries, Mishor Yamin, 86800, Israel

**Keywords:** texture, pole figures, inverse pole figures, orientation distribution function, ODF, *MTEX*

## Abstract

Knowledge of the appearance of texture components and fibres in pole figures, in inverse pole figures and in Euler space is fundamental for texture analysis. For cubic crystal systems, such as steels, an extensive literature exists and, for example, the book by Matthies, Vinel & Helming [*Standard Distributions in Texture Analysis: Maps for the Case of Cubic Orthorhomic Symmetry*, (1987), Akademie-Verlag Berlin] provides an atlas to identify texture components. For lower crystal symmetries, however, equivalent comprehensive overviews that can serve as guidance for the interpretation of experimental textures do not exist. This paper closes this gap by providing a set of scripts for the *MTEX* package [Bachmann, Hielscher & Schaeben (2010). *Solid State Phenom.* **160**, 63–68] that allow the texture practitioner to compile such an atlas for a given material system, thus aiding orientation distribution function analysis also for non-cubic systems.

## Abstract 2

Knowledge of the appearance of texture components and fibres in pole figures, in inverse pole figures and in Euler space is fundamental for texture analysis; an *MTEX*-based tool is presented to predict their appearance to enable subsequent quantitative texture analysis.

## 1. Introduction

Quantitative texture analysis is concerned with detailed analysis of the orientation distribution function (ODF), *e.g.* measuring weight fractions of texture components or texture fibres. These components in turn allow one to deduce, for example, whether an observed change in the microstructure during a thermo-mechanical processing step is due to deformation, recrystallization *etc*. as each of these processes develops typical texture components. For cubic materials, such as steels, these texture components are very well understood, and a large body of research exists defining the typical components as well as identifying the processes during which they develop. For example, Ray *et al.* (1994 ▸) and Hoelscher *et al.* (1991 ▸) provide reviews of texture components for steel. Hu (1974 ▸) provided a condensed review for polycrystalline metals and alloys. Typical pole figures (PFs), inverse pole figures (IPFs), and, more importantly, locations of components and fibres in ODF plots, *i.e.* orientation densities in Euler space, are provided in these papers. Matthies *et al.* (1987 ▸) compiled standard distributions for which the computed ODFs, IPFs and PFs for various components with varying half-width of the distribution are displayed. This book provides an atlas that aids texture analysis of cubic systems, as experimentally observed ODFs can be divided into their components for further quantitative analysis. Without the knowledge of these components, quantitative texture analysis is not possible and at best a qualitative comparison of PFs or ODF sections can occur.

For non-cubic systems, however, typical texture components are material specific and much less well documented. In their overview paper, Wang & Huang (2003 ▸) attempt to overcome this situation for hexagonal crystal systems. For other crystal symmetries, to the best of our knowledge, such reviews of typical texture components do not exist except for specific systems such as quartz (Law, 2014 ▸). This limitation prevents in many cases a quantitative texture analysis as well as a deeper assignment of observed texture components to thermomechanical processes in non-cubic materials. This has become particularly evident with the development of modern large-scale neutron and synchrotron user facilities in combination with sophisticated data analysis tools – especially Rietveld analysis tools – enabling texture analysis [such as *MAUD* (Lutterotti *et al.*, 1997 ▸) or *GSAS* (Von Dreele, 1997 ▸)]. These resources allow measurement of textures of multi-phase systems, as well as low-crystal-symmetry materials in the bulk that are inaccessible or at least difficult to access by PF measurements with laboratory X-ray diffractometers or electron diffraction. But because of this limitation, the vast majority of applications of texture analysis for non-cubic materials at best report PFs. Only very rarely is a quantitative texture analysis presented, although this is almost standard for cubic materials.

The ‘real-life’ texture of a given material is typically a combination of several texture components. Knowledge of the individual texture components enables one to identify the components present in an experimental texture, thus allowing quantitative texture analysis, *e.g.* determining the weight or volume fraction of a component, to ultimately interpret how a given texture correlates with the material processing history.

In this paper, we present a tool that overcomes the limitation of needing to have a comprehensive atlas of texture components available for a given system by providing short scripts for the open-source toolbox for texture analysis *MTEX* (Bachmann *et al.*, 2010 ▸). *MTEX* can produce similar plots of ODF sections, IPFs and PFs to those provided by Matthies *et al.* (1987 ▸). For the texture practitioner, this means that observed texture components of the material under investigation can be compiled from the literature or estimated via texture modelling to produce an atlas of how these components occur in ODF sections, IPFs and PFs. Once the components are identified visually, either their volume fraction can be directly refined with *MAUD* (using the ‘Standard Functions’ representation of the ODF in *MAUD*) or the volume fractions can be calculated (*e.g.* by using the ‘volume’ command in *MTEX*) to develop a deeper understanding of microstructural changes as a function of thermomechanical treatment of a material. While *MTEX* is also a texture analysis tool similar to *popLA* (Kallend *et al.*, 1991 ▸), *BEARTEX* (Wenk *et al.*, 1998 ▸) and *LABOTEX* (Pawlik & Ozga, 1999 ▸), utilizing the powerful MATLAB (The MathWorks Inc., Natick, MA, USA) graphing capabilities allows this kind of plot to be readily produced with *MTEX*. This paper provides a brief introduction to *MTEX*, describes the scripts used to produce the ODF, PF and IPF plots, and gives examples for cubic, hexagonal and orthorhombic unit cells.

## 2. *MTEX* texture analysis toolbox

*MTEX* (Bachmann *et al.*, 2010 ▸) is a free MATLAB toolbox for texture analysis and interpretation, which provides a variety of texture analysis tools. *MTEX* can be downloaded from http://mtex-toolbox.github.io/download.html and allows users to produce different visualizations of a given texture component: PF and IPF as well as sections of Euler space showing the orientation density. A PF is a stereographic projection of the orientation distribution function showing the resulting pole densities of the plane normals of a single crystallographic lattice plane for all sample directions in the coordinate system of the sample. An IPF is a stereographic projection of the pole density of all crystal lattice plane normals for a single sample direction. Since both of these visualizations are projections of the ODF, information is lost, and a more complete visualization of the ODF is provided by sections through Euler space, showing the frequency or probability of all possible crystal orientations in the sample coordinate system (Suwas & Ray, 2014 ▸; Kocks *et al.*, 2000 ▸; Randle & Engler, 2014 ▸).

In order to plot PFs, IPFs and ODFs in *MTEX*, crystal symmetry and specimen symmetry need to be defined. A texture component, *e.g.* a single orientation or a fibre, is defined in the specimen coordinate system. The mathematical description of texture components in *MTEX* is accomplished by kernel functions, and one of the available functions needs to be chosen. Finally, *MTEX* offers five types of texture components, two of which, the unimodal and fibre components, will be used here. In the following sections, more detail for each of the aforementioned parameters is provided to allow the reader to understand the examples, followed by a short example of the corresponding *MTEX* command. Complete descriptions of these commands are given in the *MTEX* documentation.

### 2.1. Crystal symmetry

Texture analysis requires solely the point group of the crystal symmetry. In *MTEX*, however, the crystal symmetry may be defined by the space group, point group or Laue group of the crystal. The crystal symmetry is stored in *MTEX* as a variable of the type crystalSymmetry. Command 1 shows an example of the definition for hexagonal α-Ti. The parameters of the crystalSymmetry object in the form used below are point group, lattice parameters and alignment of the crystal axes with the Euclidean crystal coordinate system. The result of this command is assigned to the variable CS.

Command 1: CS = crystalSymmetry(’6/mmm’, [2.9356 2.9356 4.689], ’X||a*’, ’Y||b’, ’Z||c’);

### 2.2. Specimen symmetry

The specimen symmetry can add symmetry due to thermomechanical processes, *e.g.* the fibre symmetry around the deformation axis from an extrusion process or uni-axial compression, or the mirror symmetry of a rolling process. Therefore, specimen-symmetry definition can affect the Euler space minimal dimension. For the general case, when the sample has no symmetry or is misaligned with respect to the symmetry of the texture, the full range of Euler space needs to be considered in order to represent all possible orientations. Any other sample symmetry will reduce the Euler space. For example, if a sample was deformed by rolling and is perfectly aligned with the instrument coordinate system, orthorhombic symmetry can be assumed and the Euler space will be reduced. Command 2 illustrates two different ways to define triclinic specimen symmetry (the general case). The result of this command is assigned to the variable SS.

Command 2: SS = specimenSymmetry(’1’) or SS = specimenSymmetry(’triclinic’)

### 2.3. Orientation

The weight, or probability, of all possible orientations describes the texture of the sample. Any orientation can be described by three rotation angles, the Euler angles, which may be defined using conventions given by Bunge (1982 ▸), Roe (1965 ▸) and Matthies *et al.* (1987 ▸). Furthermore, orientations can be given by pairs of Miller indices and crystal directions, {*hkl*}〈*uvw*〉 or {*hkil*}〈*uvtw*〉 for hexagonal crystal symmetry, indicating for example a set of lattice plane normals {*hkil*} parallel to the sample normal direction (ND) and a family of crystal directions parallel to the rolling direction (RD) of the sample. Command 3 shows orientations for which the {0001} plane normals are parallel to ND and the 〈10 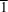 0〉 directions are parallel to RD. In this command ND and RD are determined by a sequence of lattice vectors.

Command 3: o = orientation.byMiller([0 0 1],[1 0 0],CS,SS)

### 2.4. Kernel function

**Figure 1:**
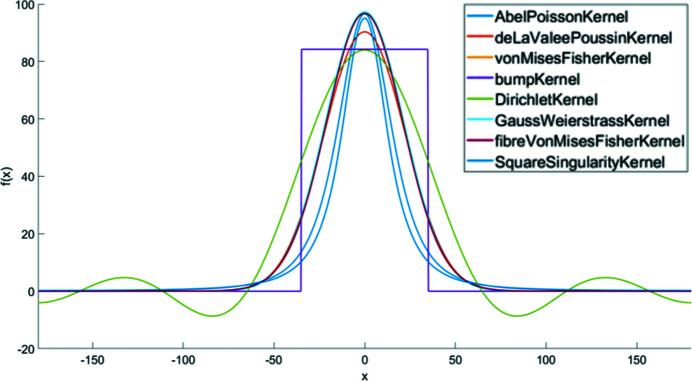
The PDFs for all kernel functions in *MTEX*.

In polycrystalline materials, texture components do not appear as single orientations but have a certain variance. This is commonly modelled by bell-shaped density functions like the Gaussian. As there is no Gaussian in orientation space, several generalizations such as the Abel–Poisson kernel, the Gauss–Weierstrass kernel, the von Mises–Fisher kernel and the de la Vallée Poussin kernel have been suggested in the literature for this purpose. In practice, all these kernel functions behave quite similarly and the choice of a specific kernel function is much less important than the choice of its half-width. In this work all standard components are modelled using the de la Vallée Poussin kernel, which is the default choice in *MTEX*. Fig. 1 ▸ shows the probability distribution functions (PDFs) for all kernel functions existing in *MTEX*. The script that produces this figure is described in Appendix *A* [App appa] and the *MTEX* documentation.

## 3. Visualization of components

The previously introduced concepts are applied to visualize texture components in *Standard Distributions in Texture Analysis* (Matthies *et al.*, 1987 ▸) for cubic crystal symmetry. To establish the compatibility of the *MTEX* scripts presented here with this standard work, we choose the {011}〈100〉 cubic component (the Goss component), which is typical of recrystallization textures in face- (f.c.c.) and body-centred cubic (b.c.c.) crystal systems and is shown on pages 278–323 of Volume 1 of *Standard Distributions in Texture Analysis*.

Having established the reproducibility for cubic systems (Section 3.1), we extend the concept to non-cubic crystals (Sections 3.2 and 3.3). In non-cubic crystals (*e.g.* hexagonal) the components strongly depend on the lattice parameter ratio (*e.g.* *c*/*a*) of a given material and therefore are not readily covered by the literature. Striving to review common texture components, we show the basal fibre component of the hexagonal α phase in Ti–6Al–4V (see Section 3.2), which results from rolling (Philippe, 1994 ▸). We note in passing that a splitting of the (0001) pole maxima can occur for basal fibres in hexagonal cubic packed (h.c.p.) materials depending on the *c*/*a* ratio in the presence of twinning (Wang & Huang, 2003 ▸). The second example in a hexagonal unit cell shows the {10 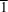 3}〈 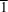 2 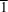 0〉 component, also occurring in hexagonal materials, which is known as the recrystallization component in titanium alloys (Wagner *et al.*, 2002 ▸). Finally, in order to illustrate the ability of *MTEX* to deal with all crystal symmetries, we chose to plot the {001}〈010〉 component in orthorhombic olivine (space group *Pbnm*) (see Section 3.3).

### 3.1. Goss component in a cubic system

**Figure 2:**
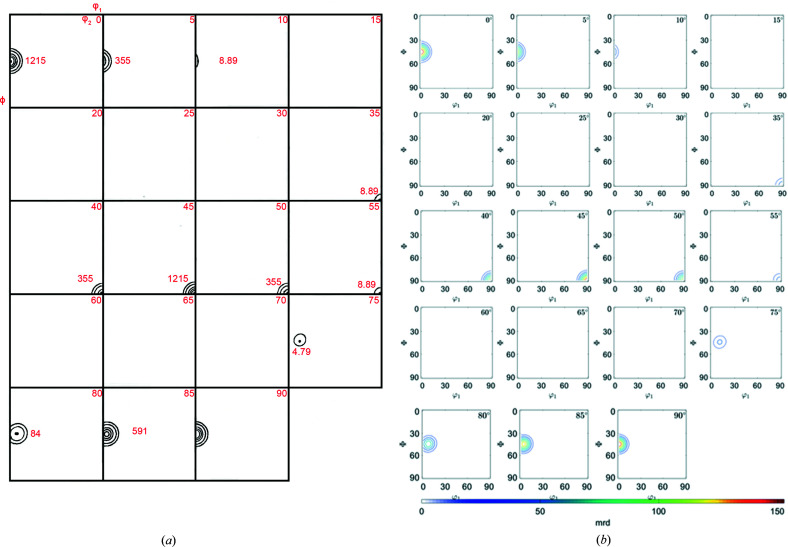
ODF maps for the Goss component with 7.5° half-width: (*a*) schematic representation after Matthies *et al.* (1987 ▸) and (*b*) produced by an *MTEX* script.

The script visualizing the Goss component {011}〈100〉, typical for f.c.c. and b.c.c. crystals and known as recrystallization texture (Hu, 1974 ▸), using the de la Vallee Poussin kernel (default function in *MTEX*) with a half-width of 7.5° is described in Appendix *B* [App appb]. Figs. 2 ▸(*b*), 3 ▸(*b*) and 4 ▸(*b*) show the resulting ODF maps, the IPFs along the rolling [100], normal [001] and transverse [010] directions, and the (110), (311), (111) and (100) PFs, respectively. While the plots are not identical, owing to the different mathematical representations, the agreement between the spherical harmonics representation of Matthies *et al.* (1987 ▸) [Figs. 2 ▸(*a*), 3 ▸(*a*) and 4 ▸(*a*)] and the plots produced by *MTEX* establishes that the parameters chosen for the plots with *MTEX* are suitable.

### 3.2. Hexagonal unit cell

As an example of a non-orthogonal unit cell we show the basal fibre and recrystallization component for the Ti crystal structure.

#### 3.2.1. Texture fibre in a hexagonal system

**Figure 5:**
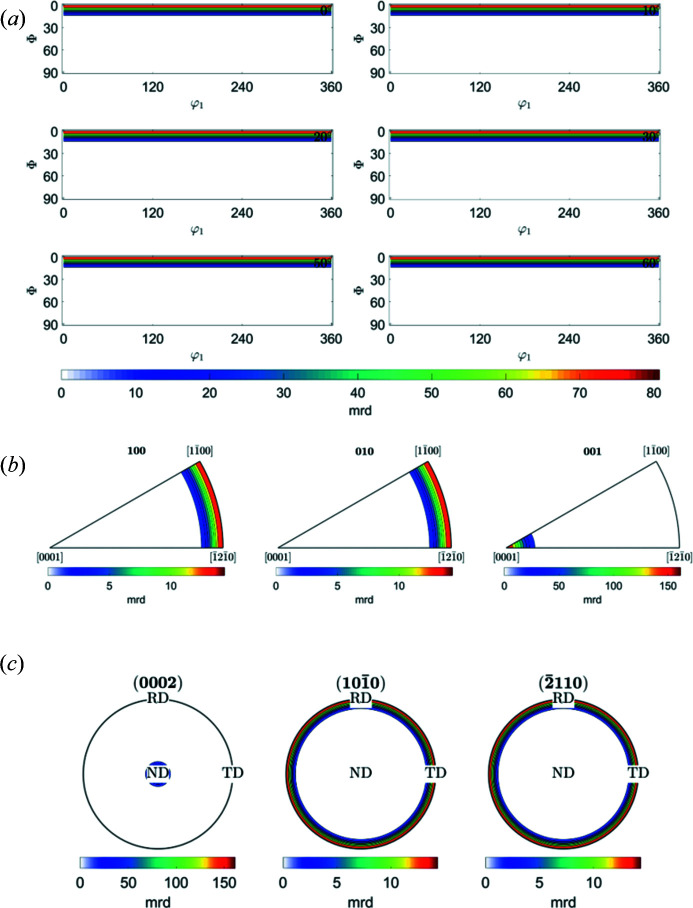
*MTEX* plots of (*a*) ODF maps, (*b*) IPFs in RD (100), TD (010) and ND (001) directions, and (*c*) calculated PFs (0002), (10 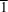 0) and ( 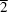 110) of fibre component {0001} in a hexagonal crystal parallel to ND.

Fig. 5 ▸ displays (*a*) ODF maps, (*b*) IPFs, and (*c*) (0002), (10 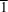 0) and ( 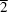 110) PFs for the basal fibre (0001) parallel to the ND direction. The script is displayed and explained in Appendix *C* [App appc]. The results of the plots agree with Fig. 1(*a*) (PFs) and Fig. 5(*a*) (ODFs) of Wang & Huang (2003 ▸).

#### {10 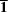 3}( 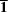 2 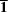 0) component in a hexagonal unit cell

3.2.2.

**Figure 6:**
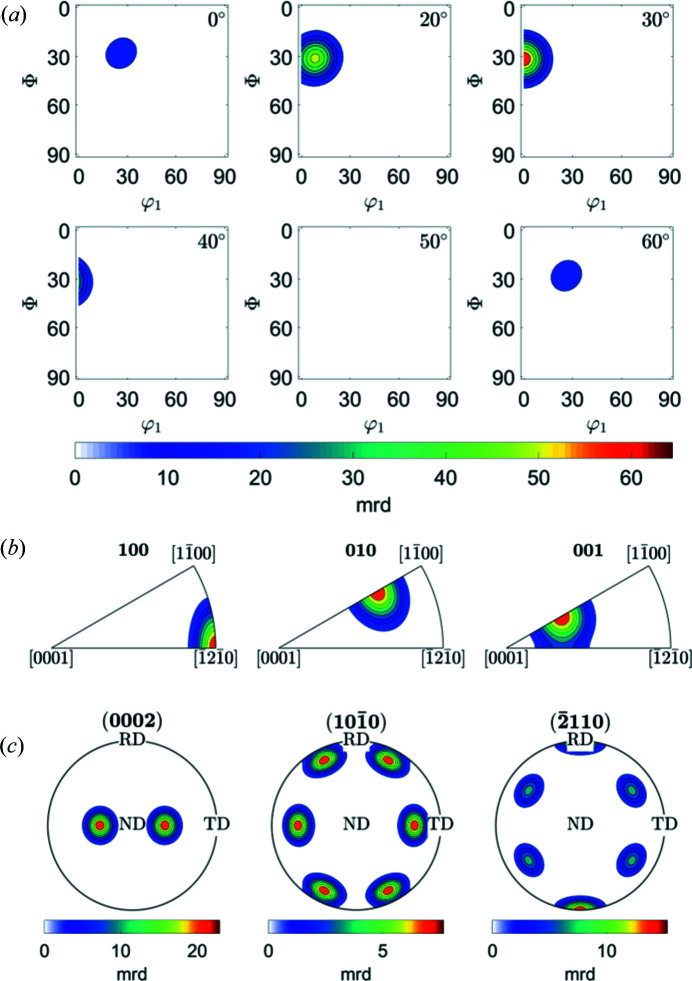
*MTEX* plots for (*a*) ODF maps, (*b*) IPFs in RD, TD and ND directions, and (*c*) calculated (0002), (10 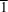 0) and ( 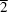 110) PFs of the {10 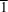 3}〈 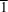 2 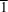 0〉 component in a hexagonal crystal for *c*/*a* = 1.597.

The following example shows Euler space sections, IPFs and PFs for the {10 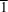 3}〈 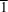 2 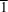 0〉 component, which is known as the recrystallization component in hexagonal systems (Wagner *et al.*, 2002 ▸). The *MTEX* script to produce plots like Fig. 6 ▸ is provided in Appendix *D* [App appd].

**Figure 7:**
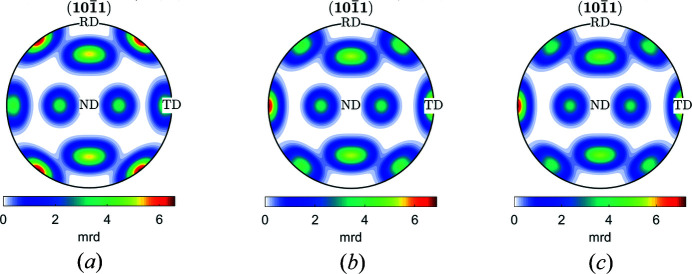
*MTEX* plots for (10 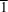 1) PFs of {10 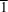 3}〈 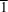 2 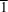 0〉 with (*a*) *c*/*a* > ideal, (*b*) *c*/*a* = ideal and (*c*) *c*/*a* < ideal.

**Figure 8:**
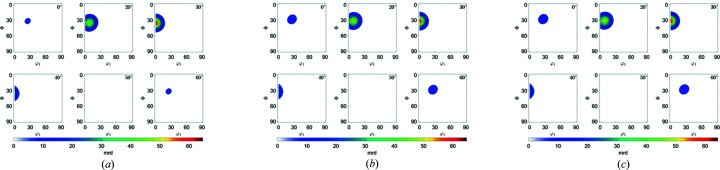
*MTEX* plots for ODFs of {10 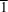 3}〈 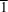 2 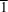 0〉 with (*a*) *c*/*a* > ideal, (*b*) *c*/*a* = ideal and (*c*) *c*/*a* < ideal.

The *c*/*a* ratio of h.c.p. materials can influence the ODFs, IPFs and PFs. Figs. 7 ▸(*a*)–7 ▸(*c*) show a comparison of the {10 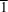 3}〈 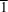 2 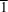 0〉 components for values of *c*/*a* above, equal to and below the ideal *c*/*a* ratio of 1.633 in the (10 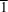 1) PF, respectively. Zn has lattice parameters *a* = 2.665 Å, *c* = 4.947 Å and *c*/*a* = 1.856, which is above the ideal ratio. Mg has lattice parameters *a* = 3.210 Å, *c* = 5.210 Å and *c*/*a* = 1.623, close to the ideal ratio. Ti has lattice parameters *a* = 2.951 Å, *c* = 4.683 Å and *c*/*a* = 1.5787, which is below the ideal ratio. The difference can be observed clearly in the PFs and to a lesser extent in the ODFs [Figs. 8 ▸(*a*)–8 ▸(*c*)]. Note that the *c*/*a* ratio has no influence on the (10 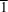 0) and (0001) PFs (not shown).

### 3.3. Orthorhombic unit cell

**Figure 9:**
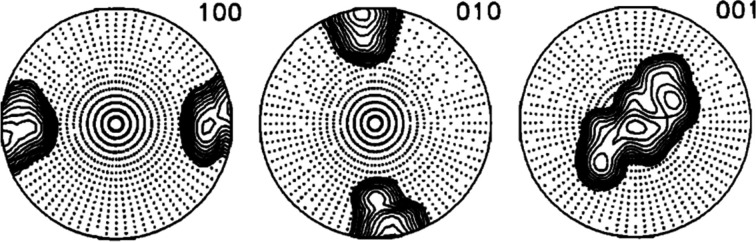
Simulated PFs for olivine during deformation and recrystallization (Wenk & Tomé, 1999 ▸).

**Figure 10:**
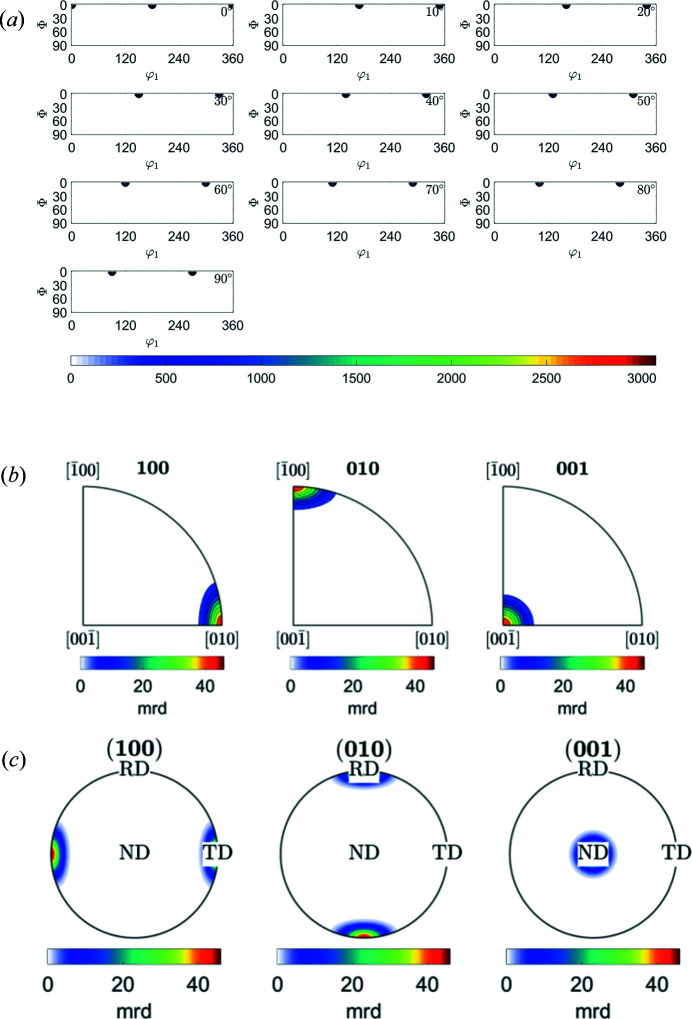
*MTEX* plots of (*a*) ODF maps, (*b*) IPFs in RD, TD and ND directions, and (*c*) {100}, {010} and {001} PFs of the {001}〈010〉 component in the olivine crystal.

The texture of low-symmetry crystal systems is rarely analyzed in terms of weight fractions of components, as is the case, in particular, for steel textures. As mentioned above, the relative simplicity of texture component reconstruction using *MTEX*, even for low-symmetry structures, could significantly help with such quantitative analysis. Olivine is an example of that. Wenk & Tomé (1999 ▸) applied a deformation-based model for dynamic recrystallization to the prediction of texture and microstructure development of olivine deformed in simple shear to large strains (Fig. 9 ▸). The script visualization of the {001}〈010〉 component for the orthorhombic unit cell of olivine is shown in Appendix *E* [App appe]. In this example we show the ODF, IPF and PF in *MTEX* without the tilt observed in Fig. 9 ▸. The orientation assigned to variable *o* is given in the Bunge Euler-angle convention. From Fig. 9 ▸ we can conclude that the Euler angles for this texture component are φ_1_ = 0, Φ = 0, φ_2_ = 0. *MTEX* is able to calculate an {*hkl*}〈*uvw*〉 component from a given orientation provided in Euler angles. The last six lines in Appendix *E* [App appe] are given to calculate {*hkl*} and 〈*uvw*〉 for the φ_1_ = 0, Φ = 0, φ_2_ = 0 orientation. The resulting orientation in {*hkl*}〈*uvw*〉 is {001}〈010〉, which is somewhat obvious for the PFs presented in Fig. 9 ▸ but is much more complicated in the general case. Fig. 10 ▸ represents the *MTEX* result for (*a*) ODF sections, (*b*) IPFs and (*c*) PFs of the {001}〈010〉 component in olivine.

**Figure 11:**
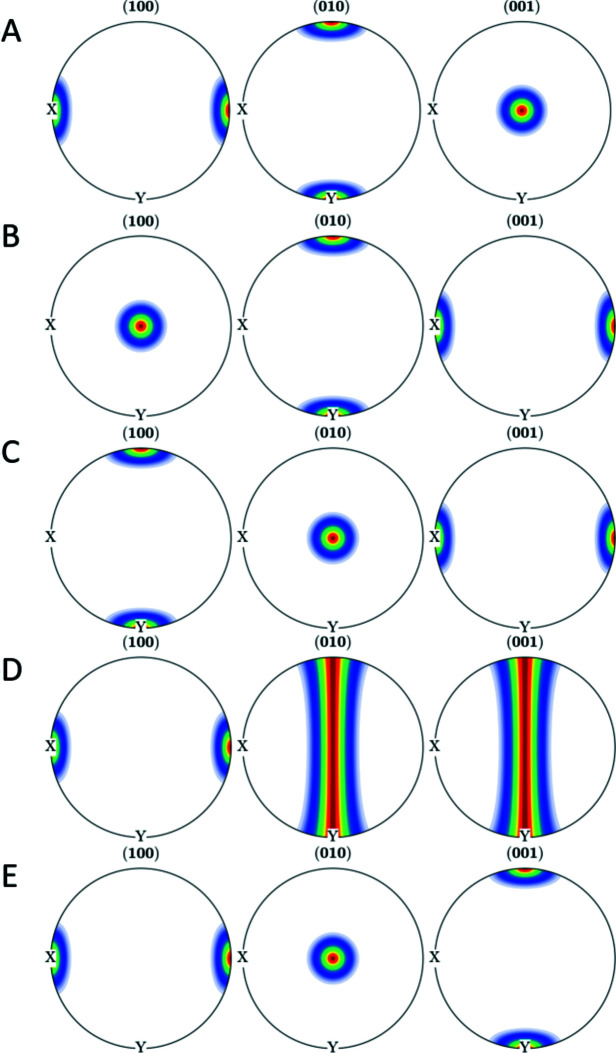
The A to E texture components of olivine displayed as pole figures.

Geoscience literature often describes olivine textures with types A to E, as described for example by Jung (2017 ▸). The script in Appendix *F* [App appf] shows an example of visualization of the A to E texture component types of olivine in terms of Miller indices. While the olivine texture component of type D is represented by a fiber, the other component types are reproduced by components of the form {*hkl*}[*uvw*]. Fig. 11 ▸ shows the pole figures of A to E texture components (Jung, 2017 ▸) and can be directly compared with Fig. 6 therein.

## 4. Summary and conclusions

We have provided scripts for the *MTEX* toolbox that provide a fast and convenient way of plotting PFs, ODFs as sections in Euler space and IPFs for a given texture component or fibre to establish where pole densities or orientation densities for this component or fibre occur. The scripts produce plots that agree with equivalent plots from standard references (Matthies *et al.*, 1987 ▸; Wang & Huang, 2003 ▸). Examples for hexagonal and orthorhombic crystal structures are given. Similar to the standard reference for cubic systems (Matthies *et al.*, 1987 ▸), such plots can help to identify texture components present in experimental textures and, for example, to assign them to specific thermomechanical processes in experimental and theoretical studies of materials with non-cubic crystal structures. Contrary to texture identification in cubic materials, where a vast literature of components (*e.g.* γ or α fibre, cube, Goss) and their origin (*e.g.* rolling, recrystallization) exists, identification of texture components in non-cubic systems is rarely reported. This tool, in combination with components reported in the literature, can help the texture researcher to overcome this limitation by producing an ‘atlas’ of the appearance of individual components for a given material. This allows determination of volume fractions in the next step, thus enabling truly quantitative texture analysis for non-cubic systems as demonstrated, for example, for Ti by Lonardelli *et al.* (2007 ▸).

## Figures and Tables

**Figure 3:**
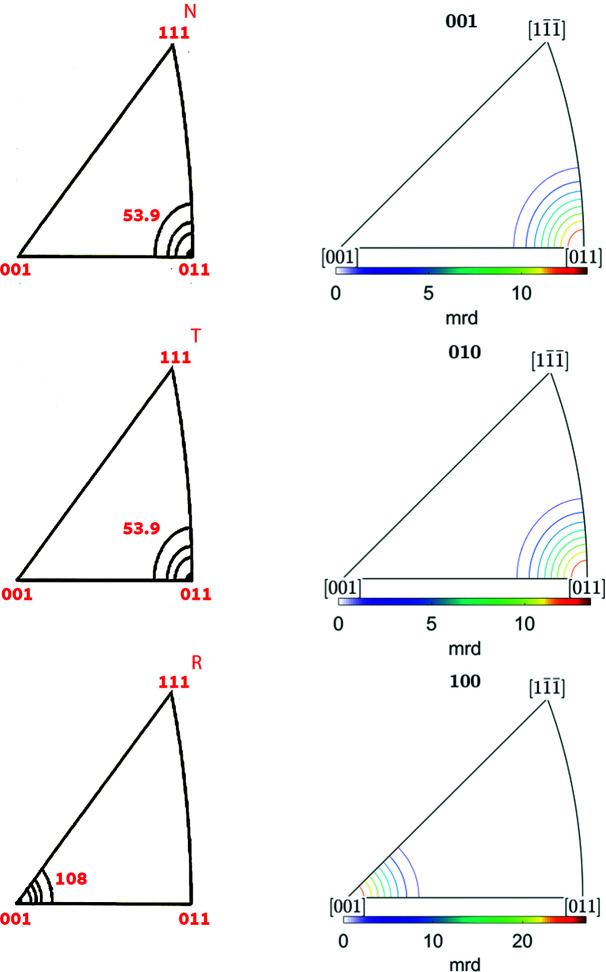
IPFs for the Goss component with 7.5° half-width along the normal direction (labelled as N or 001), transverse direction (T, 010) and rolling direction (R, 100): (*a*) schematic representation after Matthies *et al.* (1987 ▸) and (*b*) produced by *MTEX*.

**Figure 4:**
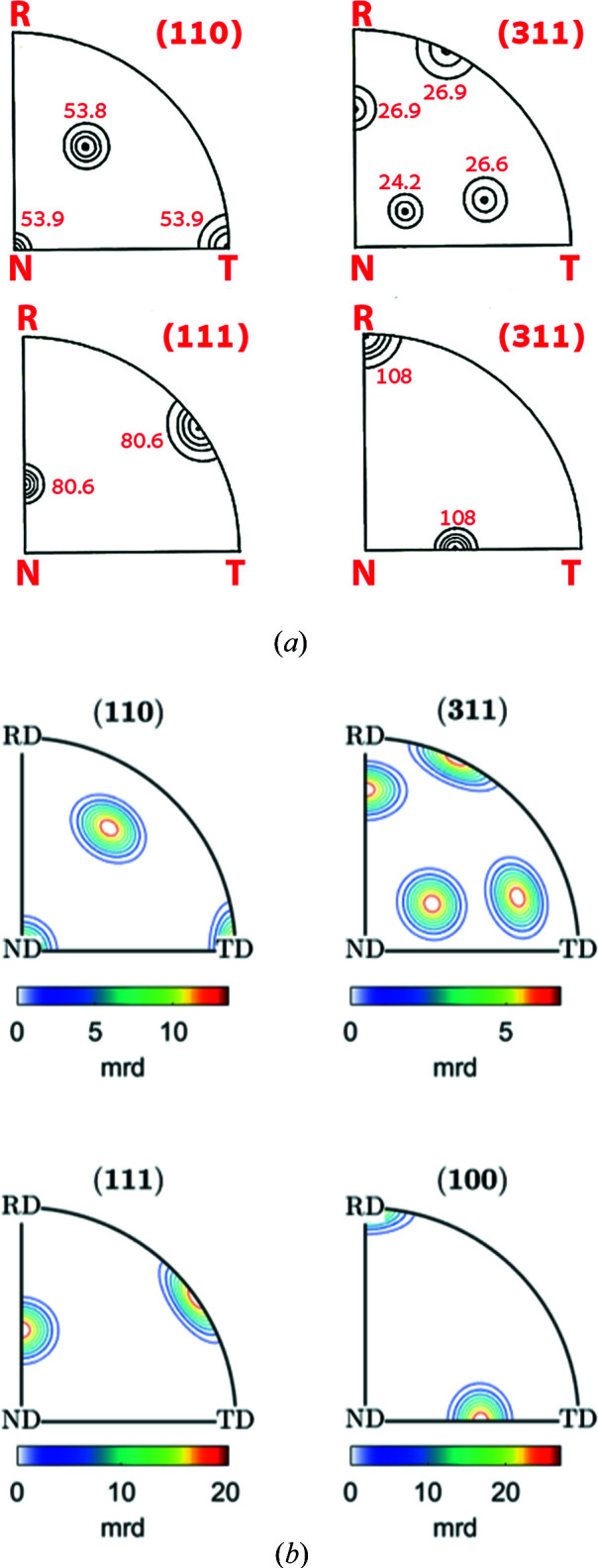
PFs for the Goss component with 7.5° half-width: (*a*) schematic representation after Matthies *et al.* (1987 ▸) and (*b*) produced with *MTEX*. Rolling, normal and transverse directions of the sample coordinate system are indicated by R, N, T in (*a*) and RD, ND, TD in (*b*).

## Appendix A

The variables psi, assigned to all possible Kernel functions in *MTEX* are assigned to an array psi. The following lines produce plots of the probability distribution function of all defined Kernel functions. The array colour defines different colours for the subsequent plotting command.

psi{1} = AbelPoissonKernel(0.79);

psi{2} = deLaValeePoussinKernel(13);

psi{3} = vonMisesFisherKernel(7.5);

psi{4} = bumpKernel(35*degree);

psi{5} = DirichletKernel(3);

psi{6} = GaussWeierstrassKernel(0.07);

psi{7} = fibreVonMisesFisherKernel(7.2);

psi{8} = SquareSingularityKernel(0.72);

close; figure(’position’,[100,100,700,450])

hold all

colour = {[0, 0.4470, 0.7410];[0.8500, 0.3250, 0.0980];...

[0.9290, 0.6940, 0.1250];[0.4940, 0.1840, 0.5560];...

[0.4660, 0.6740, 0.1880];[0.3010, 0.7450, 0.9330];...

[0.6350, 0.0780, 0.1840];...

[0.75, 0.75, 0]};

for i = 1:numel(psi)

plot(psi{i},’DisplayName’,class(psi{i}),’colour’, colour{i},’LineWidth’,2);

end

hold of f

set(findall(gcf,’-property’,’FontSize’),’FontSize’,30)

legend(gca,’show’)

xlabel(’x’)

ylabel (’f(x)’)

## Appendix B

The first command defines the crystal symmetry, lattice parameters and orientation of the crystal axes relative to the sample axes when the Euler angles are (0, 0, 0), and the sample’s symmetry is defined in the second command. The preferred axis direction is defined by the third and fourth lines. The definition of the desired texture component is assigned to the variable o.

The plot commands produce PFs, ODF sections and IPFs, respectively. Axes of PFs can be labelled using the annotate command or by changing the mtex_settings file in the *MTEX* installation directory. In this example we chose to change the mtex_settings file. Figs. 2 ▸–4 ▸ ▸ are the results of the subsequent plot commands.

CS = crystalSymmetry(’F m -3 m’, [1 1 1], ’X||a*’, ’Y||b’, ’Z||c’);

SS = specimenSymmetry(’orthorhombic’);

setMTEXpref(’xAxisDirection’,’north’);

setMTEXpref(’zAxisDirection’,’outofPlane’);

o = orientation(’Miller’,[1 1 0],[0 0 1],CS,SS)

odf = unimodalODF(o,CS,SS,’halfwidth’, 7.5*degree)

figure

plotPDF(odf,Miller({1,1,0},{3,1,1},{1,1,1},{1,0,0},CS),’antipodal’, ’contour’)

mtexColorbar(’location’,’southOutSide’,’title’,’mrd’);

set(findall(gcf,’-property’,’FontSize’),’FontSize’,30)

figure

plot(odf,’phi2’,[0 5 10 15 20 25 30 35 40 45 50 55 60 65 70 75 80 85 90]*degree,’contour’,’silent’)

set(findall(gcf,’-property’,’FontSize’),’FontSize’,30)

mtexColorbar(’location’,’southOutSide’,’title’,’mrd’);

figure

plotIPDF(odf,[xvector,yvector,zvector],’antipodal’,’contour’)

mtexColorbar(’location’,’southOutSide’,’title’,’mrd’);

set(findall(gcf,’-property’,’FontSize’),’FontSize’,30)

## Appendix C

The following script produces plots for the basal fibre {0002} parallel to the **Z** direction of the sample in hexagonal α-Ti using *MTEX*. This script shows how font size can be defined. The hkil_fibre variable is assigned to fibre orientation, from which the fibre component ODF_fibre is calculated.

CS = crystalSymmetry(’6/mmm’, [2.9356 2.9356 4.689], ’X||a*’, ’Y||b’, ’Z||c’);

SS = specimenSymmetry(’triclinic’);

setMTEXpref(’FontSize’,25);

setMTEXpref(’xAxisDirection’,’north’);

setMTEXpref(’zAxisDirection’,’intoPlane’);

hkil_fibre = Miller(0,0,0,1,CS,’HKIL’)

psi = vonMisesFisherKernel(’HALFWIDTH’,10*degree);

odf_fibre = fibreODF(hkil_fibre,zvector,SS,psi)

figure

plotPDF(odf_fibre,Miller({0,0,2},{1,0,0},{-2,1,0},CS),’antipodal’,’contourf’)

figure

plot(odf_fibre,’phi2’,[0 10 20 30 50 60]*degree,’contourf’,’silent’)

set(findall(gcf,’-property’,’FontSize’),’FontSize’,25)

figure

plotIPDF(odf_fibre,[xvector,yvector,zvector],’antipodal’,’contourf’)

set(findall(gcf,’-property’,’FontSize’),’FontSize’,30)

## Appendix D

The following script produces the {10  3}〈  2  0〉 component, a typical recrystallization component, in the hexagonal crystal symmetry for the Ti crystal structure. The specimenSymmetry in this script was set to orthorhombic, thus reducing the plot range of the Euler space sections.

CS = crystalSymmetry(’6/mmm’, [2.9356 2.9356 4.689], ’X||a*’, ’Y||b’, ’Z||c’);

SS = specimenSymmetry(’orthorhombic’);

setMTEXpref(’FontSize’,25);

setMTEXpref(’xAxisDirection’,’north’);

setMTEXpref(’zAxisDirection’,’intoPlane’);

o = orientation(’Miller’,[1 0 3],[-1 2 0],CS,SS)

odf = unimodalODF(o,CS,SS,7.5*degree)

figure

plotPDF(odf,Miller({0,0,2},{1,0,0},{-2,1,0},CS),’antipodal’,’contourf’,’upper’)

figure

plot(odf,’phi2’,[0 20 30 40 50 60]*degree,’contourf’,’silent’,)

set(findall(gcf,’-property’,’FontSize’),’FontSize’,25)

figure

plotIPDF(odf,[xvector,yvector,zvector],’antipodal’,’contourf’)

set(findall(gcf,’-property’,’FontSize’),’FontSize’,35)

## Appendix E

The following script defines the φ_1_ = 0, Φ = 0, φ_2_ = 0 component in an orthorhombic unit cell with the lattice parameters of olivine and converts the component to the {*hkl*}〈*uvw*〉 convention by computing the closest {*hkl*}〈*uvw*〉 vectors.

CS = crystalSymmetry(’P b n m’, [4.78 10.25 6.3], ’X||b’, ’Y||a’, ’Z||c’);

SS = specimenSymmetry(’1’);

figure

o = orientation(’Euler’,0*degree,0*degree,0*degree,CS,SS)

psi = vonMisesFisherKernel(’HALFWIDTH’,5*degree);

odf = unimodalODF(o,CS,SS,psi)

plotPDF(odf,Miller({1,0,0},{0,1,0},{0,0,1},CS), ‘HALFWIDTH’,5*degree, ’antipodal’)

annotate([vector3d.X,vector3d.Y,vector3d.Z],’label’,{’RD’,’TD’,’ND’},’backgroundcolor’,’w’)

figure

plot(odf,’phi2’,[0 10 20 30 40 50 60 70 80 90]*degree,’contourf’,’silent’)

figure

plotIPDF(odf,[xvector,yvector,zvector],’antipodal’,’contourf’)

hkl = o \ vector3d.Z;

hkl.dispStyle = ’hkl’;

hkl = round(hkl)

uvw = o \ vector3d.X;

uvw.dispStyle = ’UVW’;

uvw = round(uvw)

## Appendix F

The following script produces the five olivine texture components A to E. The type-D texture component of olivine, which can be described as a girdle distribution of the [010] and [001] axes, can be visualized using the fibre component.

setMTEXpref(’xAxisDirection’,’west’)

CS = crystalSymmetry(’P b n m’, [4.78 10.25 6.3], ’X||b’, ’Y||a’, ’Z||c’);

SS = specimenSymmetry(’1’);

type_A = orientation(’Miller’,[0 0 1],[0 1 0],CS,SS);

ODF_A = unimodalODF(type_A,CS,SS,7.5*degree);

plotPDF(ODF_A,Miller({1,0,0},{0,1,0},{0,0,1},CS), ‘HALFWIDTH’,5*degree, ’antipodal’)

type_B = orientation(’Miller’,[1 0 0],[0 0 1],CS,SS);

ODF_B = unimodalODF(type_B,CS,SS,7.5*degree);

plotPDF(ODF_B,Miller({1,0,0},{0,1,0},{0,0,1},CS), ‘HALFWIDTH’,5*degree, ’antipodal’)

type_C = orientation(’Miller’,[0 1 0],[0 0 1],CS,SS);

ODF_C = unimodalODF(type_C,CS,SS,7.5*degree);

plotPDF(ODF_C,Miller({1,0,0},{0,1,0},{0,0,1},CS), ‘HALFWIDTH’,5*degree, ’antipodal’)

type_D = fibreODF(Miller(1,0,0,CS),xvector);

plotPDF(type_D,Miller({1,0,0},{0,1,0},{0,0,1},CS), ‘HALFWIDTH’,5*degree, ’antipodal’)

type_E = orientation(’Miller’,[0 1 0],[1 0 0],CS,SS);

ODF_E = unimodalODF(type_E,CS,SS,7.5*degree);

plotPDF(ODF_E,Miller({1,0,0},{0,1,0},{0,0,1},CS), ‘HALFWIDTH’,5*degree, ’antipodal’)

## References

[bb1] Bachmann, F., Hielscher, R. & Schaeben, H. (2010). *Solid State Phenom.* **160**, 63–68.

[bb2] Bunge, H. J. (1982). *Texture Analysis in Materials Science: Mathematical Methods.* London: Butterworths.

[bb3] Hölscher, M., Raabe, D. & Lücke, K. (1991). *Steel Res.* **62**, 567–575.

[bb4] Hu, H. (1974). *Texture Stress Microstruct.* **1**, 233–258.

[bb5] Jung, H. (2017). *Geosci. J.* **21**, 985–1011.

[bb7] Kallend, J. S., Kocks, U., Rollett, A. & Wenk, H.-R. (1991). *Texture Stress Microstruct.* **14**, 1203–1208.

[bb8] Kocks, U. F., Tomé, C. N., Wenk, H.-R. & Beaudoin, A. J. (2000). *Texture and Anisotropy: Preferred Orientations in Polycrystals and Their Effect on Materials Properties.* Cambridge University Press.

[bb9] Law, R. D. (2014). *J. Struct. Geol.* **66**, 129–161.

[bb10] Lonardelli, I., Gey, N., Wenk, H.-R., Humbert, M., Vogel, S. C. & Lutterotti, L. (2007). *Acta Mater.* **55**, 5718–5727.

[bb11] Lutterotti, L., Matthies, S., Wenk, H.-R., Schultz, A. & Richardson, J. Jr (1997). *J. Appl. Phys.* **81**, 594–600.

[bb12] Matthies, S., Vinel, G. W. & Helming, K. (1987). *Standard Distributions in Texture Analysis: Maps for the Case of Cubic Orthorhombic Symmetry.* Akademie-Verlag Berlin.

[bb6] Pawlik, K. & Ozga, P. (1999). *Göttinger Arb. Geol. Paläontol.* SB4.

[bb13] Philippe, M. (1994). *Mater. Sci. Forum*, **157–162**, 1337–1350.

[bb14] Randle, V. & Engler, O. (2014). *Introduction to Texture Analysis: Macrotexture, Microtexture and Orientation Mapping.* Boca Raton: CRC Press.

[bb15] Ray, R. K., Jonas, J. J., Butrón-Guillén, M. P. & Savoie, J. (1994). *ISIJ Int.* **34**, 927–942.

[bb16] Roe, R. J. (1965). *J. Appl. Phys.* **36**, 2024–2031.

[bb17] Suwas, S. & Ray, R. K. (2014). *Crystallographic Texture of Materials.* London: Springer.

[bb18] Von Dreele, R. B. (1997). *J. Appl. Cryst.* **30**, 517–525.

[bb19] Wagner, F., Bozzolo, N., Van Landuyt, O. & Grosdidier, T. (2002). *Acta Mater.* **50**, 1245–1259.

[bb20] Wang, Y. N. & Huang, J. C. (2003). *Mater. Chem. Phys.* **81**, 11–26.

[bb21] Wenk, H.-R., Matthies, S., Donovan, J. & Chateigner, D. (1998). *J. Appl. Cryst.* **31**, 262–269.

[bb22] Wenk, H.-R. & Tomé, C. N. (1999). *J. Geophys. Res.* **104**, 25513–25527.

